# First records of the genera *Aclitus* and *Protaphidius* (Hymenoptera, Braconidae, Aphidiinae) from South Korea

**DOI:** 10.3897/BDJ.13.e161563

**Published:** 2025-08-08

**Authors:** Sangjin Kim, Juhyeong Sohn, Minho Lee, Hyojoong Kim

**Affiliations:** 1 Animal Systematics Laboratory, Department of Biological Science, Kunsan National University, Gunsan-si, Republic of Korea Animal Systematics Laboratory, Department of Biological Science, Kunsan National University Gunsan-si Republic of Korea; 2 Insect Biosystematics Laboratory, Department of Agricultural Biotechnology, Seoul National University, Seoul, Republic of Korea Insect Biosystematics Laboratory, Department of Agricultural Biotechnology, Seoul National University Seoul Republic of Korea; 3 Research Institute of Agricultural and Life Sciences, Seoul National University, Seoul, Republic of Korea Research Institute of Agricultural and Life Sciences, Seoul National University Seoul Republic of Korea

**Keywords:** DNA barcoding, natural enemy, parasitoid wasps, systematics, taxonomy

## Abstract

**Background:**

The aphidiine genera *Aclitus* Förster, 1863 and *Protaphidius* Ashmead, 1900 (Hymenoptera, Braconidae, Aphidiinae) each contain only a few species worldwide. In this study, we report the first records of *Aclitus* and *Protaphidius* from Korea, based on specimens of *Aclitussappaphis* Takada & Shiga, 1974 and *Protaphidiusnawaii* (Ashmead, 1906).

**New information:**

We provide detailed morphological characters, diagnostic characters, and photographic documentation for both species. A new host record, *Hamamelistesbetulinus* (de Horváth, 1896), of *Aclitussappaphis* is provided. To support species identification and future phylogenetic studies, mitochondrial (*COI*, *COII*, *ND1*, *Cytb*) and nuclear (*28SD2*, *EF1A1*, *Wingless*) gene sequences for both species are also provided.

## Introduction

The genera *Aclitus* Förster, 1863 and *Protaphidius* Ashmead, 1900 (Hymenoptera, Braconidae, Aphidiinae) are known to consist of only two and three species worldwide, respectively (Ratzeburg 1848, Starý 1959, Takada and Shiga 1974, Takada 1983, Davidian 2007, Yu et al. 2016, Farahani et al. 2017). In this study, these two genera are newly recorded from South Korea (National Institute of Biological Resources (NIBR) 2024).

The genus *Aclitus* includes two species: the Western Palaearctic species *A.obscuripennis* Förster, 1863 and the Eastern Palaearctic species *A.sappaphis* Takada & Shiga, 1974, which parasitise root aphids. *Aclitussappaphis* exhibits several morphological adaptations to a subterranean lifestyle, including erect setae covering the entire body, short and moniliform antennae, small eyes, reduced segmentation in the labial and maxillary palpi, a broadly rounded metasoma, robust legs and large, acute claws (Starý 1959, Takada and Shiga 1974). *Sappaphispiri*, its host aphid, which is primarily associated with *Pyrus* spp., migrates to the underground parts of *Artemisia* in late spring, reproduces during summer and returns to *Pyrus* sp. in autumn for overwintering. *Aclitussappaphis* completes its life cycle underground and enters diapause in the form of a mummy during the host's overwintering season (Takada and Shiga 1974). Parasitism occurs only when the host aphid is tended by ants of the genus *Pheidole* Westwood, 1839 (Hymenoptera, Formicidae) (Takada and Shiga 1974, Takada and Yoshiaki 1985). Parasitoids display synoeketic behavior, being ignored by the ants and they have been observed feeding on aphid honeydew.

Mackauer (1961) examined the shape of the female genitalia, particularly the second valvifer, as well as the ovary and the cephalic structure of the final instar larva to determine the taxonomic position of *Aclitus*. The genus is characterised by a transversely carinate propodeum, broad petiole, straight and elongated ovipositor sheath, and a fore wing vein R1 extending nearly to the wing margin — all considered plesiomorphic features.

The genus *Protaphidius* was established by Ashmead (1900) as a replacement name for *Coelonotus* Förster, 1862, which was preoccupied by *Coelonotus* (Peters, 1855) (Syngnathiformes, Syngnathidae). The type species, *Coelonotusrufus* Förster, 1862, was later recognised as a junior synonym of *Aphidiuswissmannii* Ratzeburg, 1848, resulting in the valid combination *Protaphidiuswissmannii* (Ratzeburg, 1848).

The genus *Protaphidius* consists of three species: the Western Palaearctic species *P.wissmannii* and two Eastern Palaearctic species, *P.nawaii* (Ashmead, 1906) and *P.belokobylskiji* Davidian, 2007 (Ratzeburg 1848, Takada 1983, Davidian 2007, Davidian and Gavrilyuk 2014). *Protaphidiusnawaii* was originally described from Japan under the genus *Aclitus* by Ashmead (1906), later synonymised with *P.wissmannii* by Starý (1959), but subsequently re-validated as a distinct species by Starý and Schlinger (1967). Specimens from Japan previously identified as *P.wissmannii* were later confirmed as *P.nawaii* (Takada 1983).

Species of *Protaphidius* parasitise host aphid species in the genus *Stomaphis* Walker, 1870 (Hemiptera, Aphididae, Lachninae), which exhibit a strong dependence on mutualistic ants, particularly the genus *Lasius* Fabricius, 1804 (Hymenoptera, Formicidae). For example, *Lasiusjaponicus* Santschi, 1941 protects them from natural enemies by constructing shelters from soil and woody debris to block access by predators and parasitoids (Takada 2009, Depa et al. 2015, Depa et al. 2017, Lee et al. 2022). Parasitism by *Protaphidius* species is typically observed within these ant shelters (Takada 1983, Takada 2009, Kaliuzhna 2012, Tetsuya et al. 2020). *Protaphidius* species exhibit a highly modified metasoma, with metasomal tergite 4 and the following tergites being distinctly tubiform and telescopically extendable, forming a sham ovipositor. Starý (1970) noted that, although the morphological and ecological traits of these parasitoids suggest a close affinity to the genus *Pauesia* Quilis, 1931, *Protaphidius* represents a highly isolated lineage within Aphidiinae.

## Materials and methods

### Field and Taxonomic works

*Protaphidiusnawaii* samples were collected from *Stomaphis* sp. mummies found on *Quercusmongolica* Fisch. ex Ledeb., 1850 (Fagaceae). Mummified aphids were collected from crevices in the bark of the host plant’s trunk approximately 1 m above ground level, after removing ant-constructed shelters made of soil (Fig. 4) and placed in clean insect breeding dishes (SPL Life Sciences, Korea). To ensure a sufficient number of emerging parasitoids, the dishes were maintained at room temperature in the laboratory. Emerging parasitoid wasps were monitored daily and collected using an insect aspirator. *Aclitussappaphis* was collected by a Malaise trap. After sorting the trap samples, the collected wasps were then preserved in 80% ethyl alcohol at -19℃.

The morphological identification was conducted using references from Takada and Shiga (1974), Takada (1983), Davidian (2007) and the Starý collection (Institute of Entomology, Czech Academy of Sciences (IECA), Czech Republic). The identification process began with morphological sorting of specimens with similar phenotypes using a stereomicroscope (Olympus SZX16, Leica M205C). After this initial sorting, we labeled the samples and proceeded with DNA extraction. The terminology follows Wharton et al. (1997).

Morphological identification was conducted, based on the measurement of specific characters of each specimen. For photography and characterisation, we used a LEICA DMC2900 digital camera mounted on a LEICA M205 C microscope (Leica Microsystems AG). During a visit to the Starý collection, two specimens were personally examined of *A.sappaphis*, which are not type specimens. Photographs were taken using a Canon EOS 60D digital camera mounted on a WeMacro rail (Shanghai Macro Photoelectric Co., China). Multiple images were taken at various focal heights and the image stacking process was performed using Mosaic V.2.3 (Tucsen Software) and HeliconFocus 7 (Helicon Soft). After stacking, illustrations were generated using Adobe Photoshop CS6. To determine the precise shape of morphological characters, we employed Mosaic V.2.3 (Tucsen Software) to show informative views of the specimens (Berkovitch et al. 2009). In the case of Korean specimens, they were preserved in ethanol and prepared as semi-slide specimens for imaging. We placed a spacer of sufficient height between the slide glass and the cover glass to accommodate the specimen, then positioned the specimen between the slide and cover glass with ethanol and proceeded with photography. Subsequently, they were prepared as dried specimens.

### Molecular works

Total genomic DNA was extracted using a LaboPass Tissue Kit (COSMOgenetech, Korea) following the manufacturer’s protocol with slight modifications. To maintain morphological integrity of the specimens, we adapted the “freezing method” described by Yaakop et al. (2013). Our modification involved extending the incubation period from 30 minutes to 2 hours at 56°C with 200 μl of TL buffer and 20 μl of proteinase K. This adjustment allowed for effective DNA extraction, while minimising damage to the specimens’ structure. Each sample underwent individual genomic DNA extraction to ensure sample-specific results; one *A.sappaphis* specimen and one *P.nawaii* specimen were used for this procedure.

For DNA barcoding, we targeted three partial regions of nuclear genes and four partial regions of mitochondrial genes. The primers used for PCR amplification of COI and other markers are listed in Table 1. These regions were amplified using the primers with AccuPower PCR PreMix (Bioneer Corp., Daejeon, Korea). The polymerase chain reaction (PCR) was performed in a 20 μl reaction mixture consisting of 3 μl of DNA extract, 2 μl of primer, and 15 μl of ddH_2_O. The PCR protocol is described in Table 2. PCR products were visualised by electrophoresis on agarose gel. When bands were observed, the samples were sent to Macrogen (Daejeon, Korea) for purification and sequencing analysis. All sequences generated in this study were deposited in GenBank.

## Taxon treatments

### Aclitus sappaphis

Takada & Shiga, 1974

47422088-F208-5B37-BA0B-852BD6EE1F94

PV646691

PV658760

PV658762

PV661833

PV662164

PV661829

PV661831

*Aclitussappaphis* Takada & Shiga, 1974

#### Materials

- **Type status:** Other material. **Occurrence:** sex: 1 female; lifeStage: adult; occurrenceID: 6F6E1534-552C-50AD-B770-135E130CBBC3; **Taxon:** scientificName: *Aclitussappaphis* Takada & Shiga, 1974; kingdom: Animalia; phylum: Arthropoda; class: Insecta; order: Hymenoptera; family: Braconidae; genus: Aclitus; specificEpithet: sappaphis; scientificNameAuthorship: Takada & Shiga, 1974; taxonRemarks: species; **Location:** higherGeography: East Asia; country: South Korea; countryCode: KR; stateProvince: Gangwon-do; municipality: Yanggu-gun; locality: DMZ Botanical Garden, Mandae-ri, Haean-myeon; **Event:** startDayOfYear: 04 Jul 2017; endDayOfYear: 18 Jul 2017; year: 2017; **Record Level:** type: Dried specimen; institutionCode: KNA, Korean National Arboterum
- **Type status:** Other material. **Occurrence:** sex: 1 female; lifeStage: adult; occurrenceID: 076AF26F-F2E1-5928-A091-E400CC6C856C; **Taxon:** scientificName: *Aclitussappaphis* Takada & Shiga, 1974; kingdom: Animalia; phylum: Arthropoda; class: Insecta; order: Hymenoptera; family: Braconidae; genus: Aclitus; specificEpithet: sappaphis; scientificNameAuthorship: Takada & Shiga, 1974; taxonRemarks: species; **Location:** higherGeography: East Asia; country: Japan; countryCode: JP; stateProvince: Hokkaido; municipality: Sapporo; **Identification:** identificationRemarks: *Amonoctonuswatanabei* (Takada, 1965) reared from *Hamamelistesbetulinus* (de Horváth, 1896) (original label: *Amonoctonuswatanabei* (Takada, 1965) reared from *Mansakiashirakabae* (Monen, 1927)); **Event:** eventDate: 08 Jun 1967; year: 1967; **Record Level:** type: Dried specimen; institutionCode: IECA, České Budějovice
- **Type status:** Other material. **Occurrence:** sex: 1 female; lifeStage: adult; occurrenceID: E995C060-5A67-5187-92B3-6DB18646BBB8; **Taxon:** scientificName: *Aclitussappaphis* Takada & Shiga, 1974; kingdom: Animalia; phylum: Arthropoda; class: Insecta; order: Hymenoptera; family: Braconidae; genus: Aclitus; specificEpithet: sappaphis; scientificNameAuthorship: Takada & Shiga, 1974; taxonRemarks: species; **Location:** higherGeography: East Asia; country: Japan; countryCode: JP; stateProvince: Hokkaido; municipality: Sapporo; **Identification:** identificationRemarks: *Amonoctonuswatanabei* (Takada, 1965) reared from *Hamamelistesbetulinus* (de Horváth, 1896) (original label: *Amonoctonuswatanabei* (Takada, 1965) reared from *Mansakiashirakabae* (Monen, 1927)); **Event:** eventDate: 08 Jun 1967; year: 1967; **Record Level:** type: Slide specimen; institutionCode: IECA, České Budějovice

#### Diagnosis

**Figure 1.**
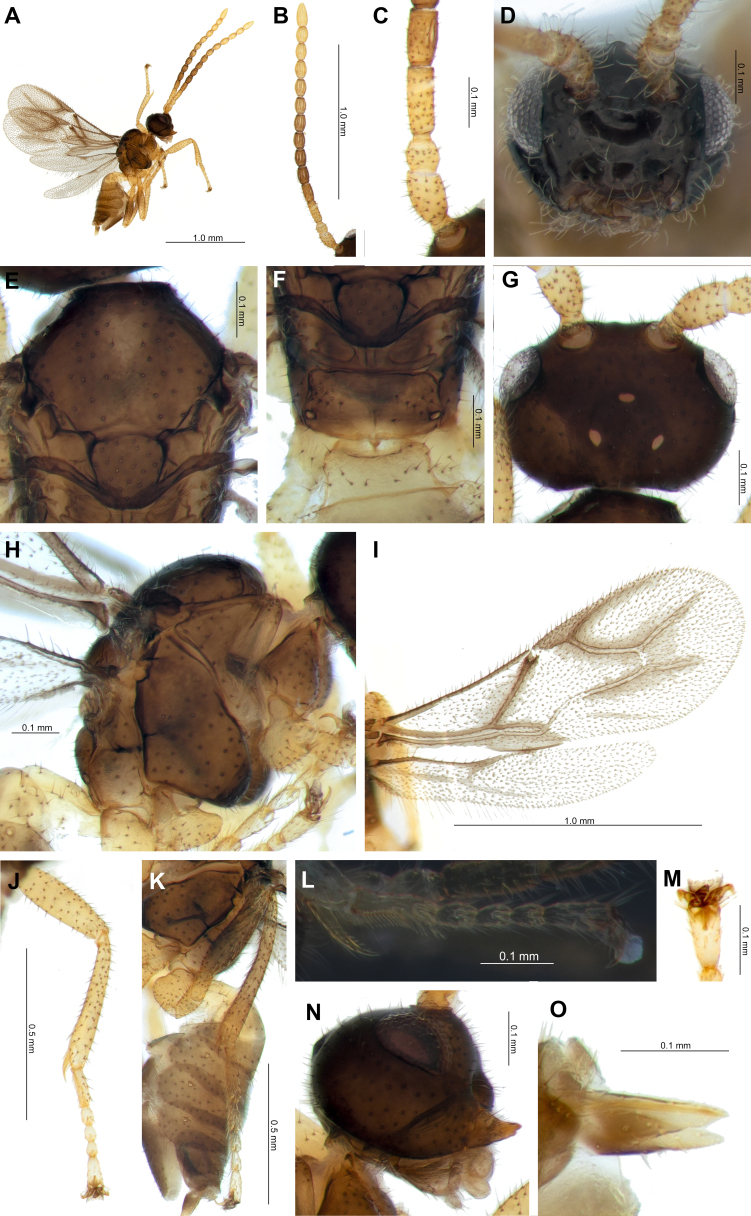
*Aclitussappaphis* female from South Korea, deposited in KNA (Korea National Arboretum): **A** Habitus, lateral view; **B** Antenna; **C** Scape, pedicel, F1 and F2; **D** Head; **E** Mesonotum; **F** Propodeum and petiole; **G** Head, dorsal view; **H** Mesosoma and petiole, lateral veiw; **I** Wings; **J** Fore leg; **K** Hind leg; **L** Libial spur with tarsus, fore leg; **M** Tarsal claws, fore leg; **N** Head, lateral view; **O** Ovipositor sheath.

**Female from South Korea.** Length of body in lateral view 3.5 mm (Fig. 1A). Length of antennae 1.6 mm (Fig. 1B) and fore wing 1.8 mm (Fig. 1I).

**Head.** Head transverse (width/median length in dorsal view = 1.4), wider than mesosoma at tegulae (width head/mesoscutum in dorsal view = 1.2) with sparse long erect hairs (Fig. 1A). Eye small, oval, sparsely setose (Fig. 1G, N). Face sparsely setose, width/height ratio 2.8 (Fig. 1D). Tentorial index 0.7 (Fig. 1D). Clypeus oval with 10 long setae. F1 longer than F2 (length F1/F2 = 1.3) (Fig. 1C). F1 and F2 2.8x and 2.2× their basal width, respectively. F1 without longitudinal placodes, F2 with three, and F3–F12 with at least four longitudinal placodes (Fig. 1B, C).

**Mesosoma.** Mesoscutum dorsal surface smooth, with 28–30 long setae along the dorsolateral part of mesoscutum each side (Fig. 1E). Scutellum without scutellar sulcus carina; nearly triangular, bearing 30 long setae (Fig. 1E). Propodeum with 10 long setae on each side, length/width of areola = 0.7 (Fig. 1F). Pterostigma (= stigma) narrow and elongated; length equal to vein R1 (= metacarpus). Vein r and 3RSa extended into center of fore wing. Veins 3RSb and 3M extended, but not reaching wing margin, vein r-m weakly sclerotised, colorless. Vein m-cu extended to center of fore wing, colorless at base. Vein 2CU developed, but colorless at base (Fig. 1I). Legs with sparse erect hairs. Fore femur maximum length/width = 3.7; fore tibia maximum length/width = 6.6, with one tibial spur; fore femur length subequal to fore tibia (length fore femur/fore tibia = 1.1); fore tarsus with five tarsomeres (Fig. 1L); fore basitarsus (= 1^st^ tarsomere) with a single row of densely comb-shaped hairs (Fig. 1L); fore basitarsus short (fore tarsus/fore basitarsus = 3.3) (Fig. 1J), claws acute (Fig. 1M). Tibia of middle and hind legs with two tibial spurs. Hind femur maximum length/width = 3.8; hind tibia maximum length/width = 7.9, hind femur shorter than hind tibia (hind femur/hind tibia length = 0.6); hind basitarsus without densely comb-shaped hairs; hind basitarsus long (hind tarsus/hind basitarsus = 2.6) (Fig. 1K).

**Metasoma.** Petiole short and transverse, widening gradually towards posterior (petiole length/width at level of spiracles = 0.9, petiole length/width at terminal = 1.3). (Fig. 1F, H). Ovipositor sheath elongately triangular with long hairs; dorsal with three and ventral with four long setae respectively (Fig. 1O).

**Color**. Antenna yellowish-brown and partly brown; scape, pedicel, F1 and F10–12 yellowish-brown, F2–9 yellowish-brown to brown, and again yellowish-brown gradually. Head, face and clypeus dark brown, mouth-parts light brown. Vein 1M, r, 3RSa, 3RSb, r-m, and m-cu vein of fore wing with brown spot. Mesosoma and metasoma dark brown; petiole and sternite 2 light brown. Legs light brown with dark apices.

#### Biology

A solitary endoparasitoid of *Sappaphispiri* [*Artemisiaprinceps*], *Hamamelistesbetulinus* (de Horváth, 1896) (in this study).

#### Notes

**Figure 2.**
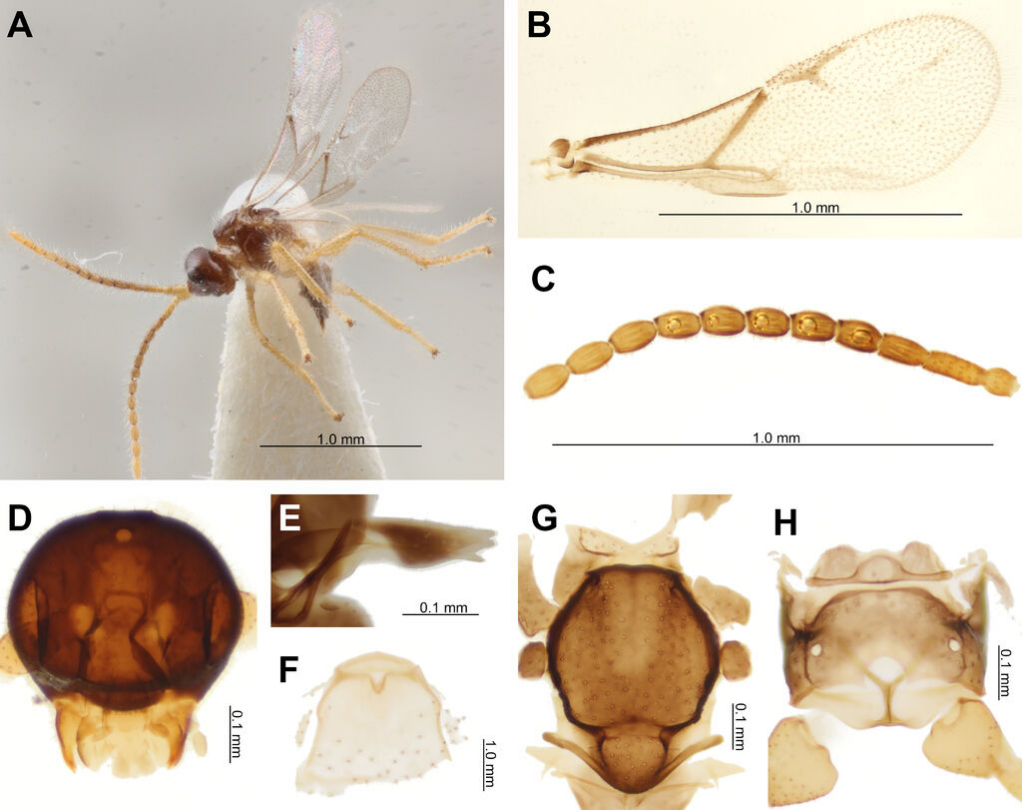
*Aclitussappaphis* female from Japan deposited in IECA, České Budějovice. **A** Habitus, lateral view, Sapporo, Hokkaido, 08.Ⅵ.1967; **B** Fore wing, 201; **C** Antennae, 201; **D** Head, 201; **E** Ovipositor sheath, 201; **F** Petiole, dorsal view, 201; **G** Mesonotum, 201; **H** Propodeum, 201.

A single row of densely comb-shaped hairs on the fore basitarsus (Fig. 1L) likely functions in removing dirt from the body, especially the head, while living underground (this character is also present in a dried specimen from Japan deposited in the Starý collection). The collector of the Japanese samples (Fig. 2A–H) most likely initially identified them as *Calaphidiuselegans* Mackauer, 1961 (= *Amonoctonuswatanabei* (Takada, 1965)), a species described two years earlier, based on the host information and collection locality. However, significant morphological differences from *C.elegans* exist, including the shape of fore wing venation, the presence of transverse areolation on the propodeum, petiole length/width ratio, and the shape of the ovipositor sheath. Although the fore wing venation of the examined specimen appears faint, this is a common issue resulting from prolonged storage in alcohol. Although the label has not been corrected, the specimen was placed within a box labeled as *Aclitussappaphis*. The original host aphis label is *Hamamelistesbetulinus* (de Horváth, 1896) (= *Mansakiashirakabae* (Monen, 1927)). Considering that the vertex outline forms an angle of nearly 90° with the frons in lateral view, the number of antennal segments, the length ratio of F1/ F2, and the length/width ratio of the pterostigma, the specimen belongs to *A.sappaphis*. A description of the type specimen can be found in Takada and Shiga (1974).

Sequences from the South Korea specimen are provided here (Suppl. material 1).

### Protaphidius nawaii

(Ashmead, 1906)

D7C77C16-7B66-5074-BCEE-17E6AAFE4B25

PV646692

PV658761

PV658763

PV661834

PV662165

PV661830

PV661832

*Aclitusnawaii* Ashmead, 1906: 188.
*Aphidiusnawaii* Watanabe, 1957: 2.
*Protaphidiuswissmannii* Starý, 1958: 88-90.
*Protaphidiusnawaii* Starý&Schlinger, 1967: 105-107.

#### Materials

- **Type status:** Other material. **Occurrence:** sex: 1 female; occurrenceID: 5B79443B-CFA4-58D8-A3E2-7B96E39AC4AB; **Taxon:** scientificName: *Protaphidiusnawaii* Ashmead, 1906; kingdom: Animalia; phylum: Arthropoda; class: Insecta; order: Hymenoptera; family: Braconidae; genus: Protaphidius; specificEpithet: *nawaii*; taxonRank: species; **Location:** higherGeography: East Asia; country: South Korea; countryCode: KR; stateProvince: Gangwon-do; municipality: Inje-gun; locality: Girin-myeon; **Identification:** identificationRemarks: reared from *Stomaphis* sp. on *Quercusmongolica* Fisch. ex Ledeb., 1850 (Fagacceae).; **Event:** eventDate: 17 Jul 2021.; **Record Level:** type: dried specimen; institutionCode: KSNU, Kunsan National University

#### Diagnosis

**Figure 3.**
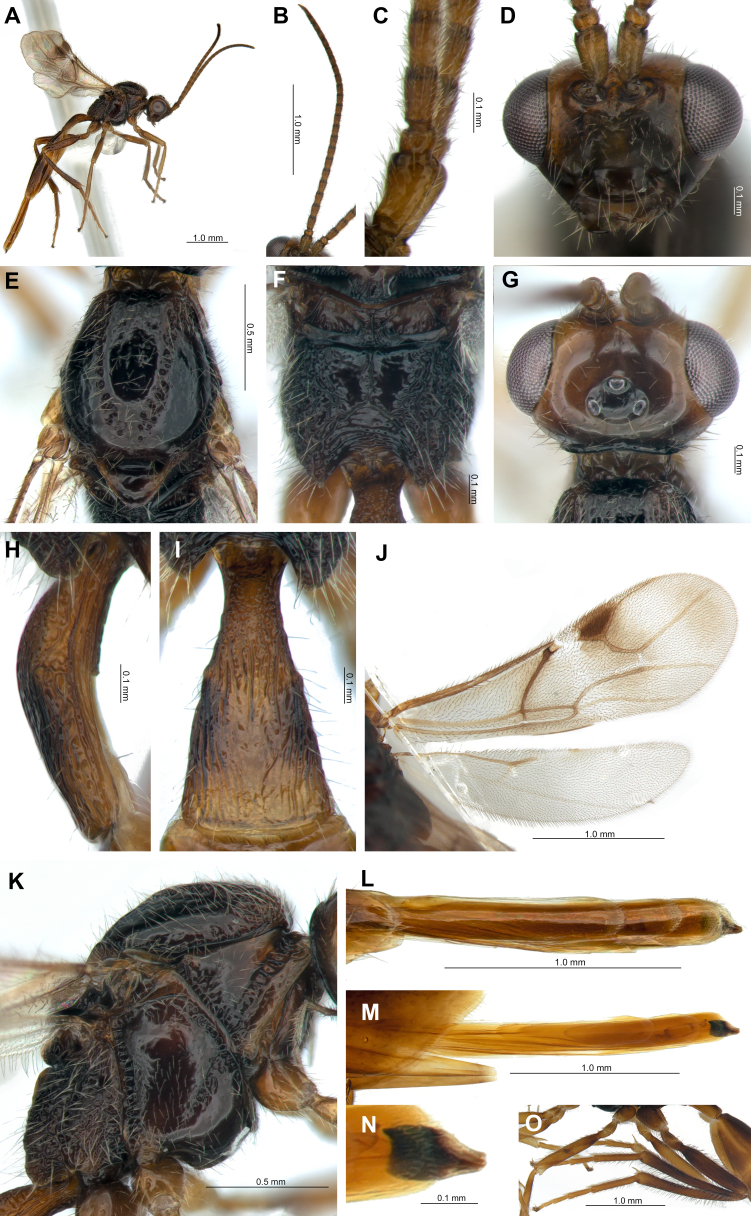
*Protaphidiusnawaii* female from South Korea deposited in KSNU (Kunsan NAtional University): **A** Habitus, lateral view; **B** Antenna; **C** Scape, pedicel, F1 and F2; **D** Head; **E** Mesonotum; **F** Propodeum; **G** Head, dorsal view; **H** Petiole, lateral veiw; **I** Petiole, dorsal view; **J** Wings; **K** Mesosoma, lateral view; **L** Metasomal tergite 4 and following tergites, dry condition; **M** Metasomal tergite 4 and the following tergites, alcohole condition; **N** Ovipositor sheath; **O** Hind leg.

**Figure 4.**
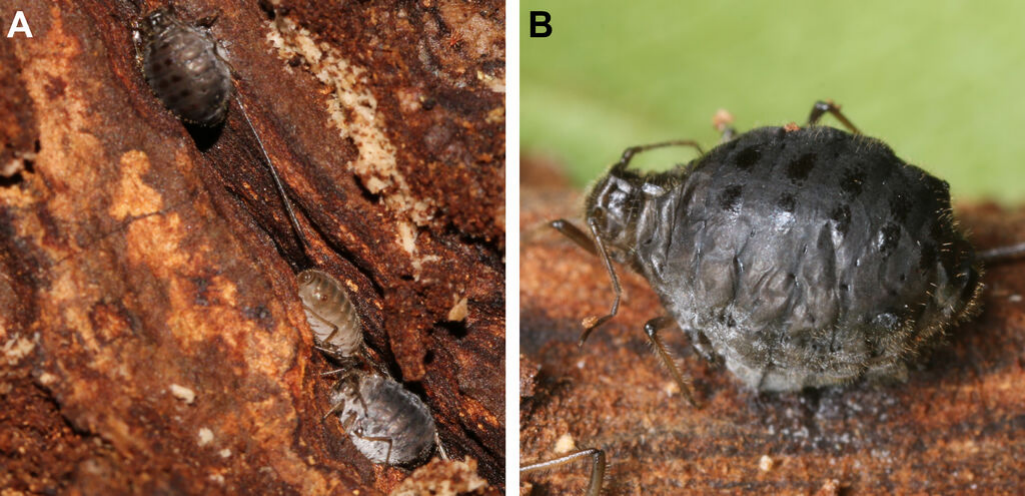
*Stomaphis* sp. occurred *Protaphidiusnawaii*. **A** live apterous viviparous female of *Stomaphis* sp.; **B** Mummy of *Stomaphis* sp.

**Female from South Korea.** Length of body in lateral view 9.3 mm (Fig. 3A). Length of antennae 2.2 mm (Fig. 3B) and fore wing 1.8 mm (Fig. 3J).

**Head.** Head transverse (width/median length in dorsal view = 2.1), with sparse long setae. Eye large (length eye/temple in dorsal view = 2.6), oval, sparsely setose (Fig. 3G). Face with sparse setae, width/height ratio 2.1 (Fig. 3D). Tentorial index 0.7 (Fig. 3D). Clypeus oval with 9 long setae. Malar space 0.5 times as long as longitudinal eye diameter (Fig. 3D). Antenna 25-segmented (Fig. 3B). F1 equal to F2 (Fig. 3C). F1 and F2 1.6x and 1.7× their basal width, respectively. F1 and F2 with five longitudinal placodes (Fig. 3C). Maxillary palp with four palpomeres, labial palp with three palpomeres (Fig. 3D).

**Mesosoma.** Mesopleuron with precoxal sulcus (= prepectal carina) at anterior half (Fig. 3K). Dorsal surface of mesoscutum sparsely setose along the dorsolateral part on each side (Fig. 3E). Scutellum without scutellar sulcus carina (Fig. 3E); nearly triangular, bearing 6 long setae (Fig. 3E). Propodeum transverse (Fig. 3F). Pterostigma broad (pterostigma length/width = 2.5), anterior third of the pterostigma colorless and a vertical colorless region runs along this point on the fore wing (Fig. 3J). Pterostigma longer than vein R1 (= metacarpus) (length pterostigma/vein R1 = 1.4). Veins r and 3RSa distinctly present. Veins 3RSb and 3M extended, but not reaching wing margin, vein r-m weakly sclerotised, colorless. Vein m-cu extended to center of fore wing. Vein 2CUb apically curved upward, not reaching wing margin (Fig. 3J). Legs sparsely setose. Fore femur maximum length/width = 5.5; tibia maximum length/width = 8.4, with one tibial spur; fore femur length equal to fore tibia; fore basitarsus long (fore tarsus/fore basitarsus = 2.3). Tibia of middle and hind legs with two tibial spurs. Hind femur maximum length/width = 5.0; hind tibia maximum length/width = 11.5, hind femur shorter than hind tibia (hind femur/hind tibia length = 0.6); hind basitarsus long (hind tarsus/hind basitarsus = 2.3) (Fig. 3O).

**Metasoma.** Petiole elongated (petiole length/width at level of spiracles = 3.1) (Fig. 3H, I), curved in lateral view; anterior and lateral parts of petiole rugose, median-dorsal part with vertical lines (Fig. 3H, I); metasomal tergites 2 and 3 fused; metasomal tergite 4 and the following tergites are distinctly tubiform and telescoped (Fig. 3L, M). Ovipositor sheath slightly downwards (Fig. 3N).

**Color.** Antenna brown and tips of each flagellomere dark brown. Head brown; face, clypeus and mouth-parts dark brown. Both anterior and posterior ends of pterostigma with vertical colorless region on fore wing. Mesosoma and dorsal view of metasoma dark brown; petiole brown, with dark brown spot behind of spiracles on each side. Metasomal tergite 4 and the following tergites brown. Legs brown with dark apices.

#### Biology

A solitary endoparasitoid of *Stomaphis* sp. [*Quercusmongolica*] (in this study), *S.aphananthae* [*Aphanantheaspera*], *S.japonica* [*Quercusacutissima*], *S.maloti* [*Mallotusjaponicus*], *S.yanonis* [*Celtissinensis*, *Zelkovaserrata*].

#### Notes

We examined several specimens of *P.wissmannii* at the Naturalis Biodiversity Center (Leiden, Netherlands), but due to image quality issues, these were not included in this paper. According to Yamamoto et al. (2020) and Staverløkk et al. (2022), the morphological diversity and species boundaries within the group remain unclear. Further studies incorporating molecular techniques, along with assessments of morphological variation and ecological traits, are needed to investigate the existence of potential cryptic species. A re-description of the type specimen can be found in Starý and Schlinger (1967).

Sequences from the South Korea specimen are provided here (Suppl. material 2).

## Supplementary Material

XML Treatment for Aclitus sappaphis

XML Treatment for Protaphidius nawaii

A4CFD404-3E88-51A0-97CC-4D0B7CEEAA75 10.3897/BDJ.13.e161563.suppl1 Supplementary material 1 Aclitussappaphis sequences Data type sequence file Brief description Sequences of Aclitussappaphis (28SD2, COI, COII, Cytb, EF1-a, ND1, Wg). File: oo_1355694.txt https://binary.pensoft.net/file/1355694 Sangjin Kim

BAA377AE-009F-544F-9274-774469E1EA85 10.3897/BDJ.13.e161563.suppl2 Supplementary material 2 Protaphidiusnawaii sequences Data type sequence file Brief description Sequences of Protaphidiusnawaii (28SD2, COI, COII, Cytb, EF1-a, ND1, Wg). File: oo_1355695.txt https://binary.pensoft.net/file/1355695 Sangjin Kim

## Figures and Tables

**Table 1.** **Primer sets used for PCR amplification of seven genetic markers for DNA barcoding.** These markers were selected for their broad utility in species identification and phylogenetic inference within Hymenoptera. Primer sequences and fragment lengths are provided as originally described in the cited references.

Gene	Primer name	Sequence (5’-3’)	Fragment size (bp)	Reference
*28S D2*	28S D2 forward	GAGAGAGTTCAAGAGTACGTG	479–500	Belshaw and Quicke (1997)
	28S D2 reverse	TTGGTCCGTGTTTCAAGACGGG		Campbell et al. (1994)
*COI*	LCO1490	GGTCAACAAATCATAAAGATATTGG	658	Folmer et al. (1994)
	HCO2198	TAAACTTCAGGGTGACCAAAAAATCA		
*COII*	C2-J-3400	ATTGGACATCAATGATATTGA	260	Simon et al. (1994)
	C2-N-3661	CCACAAATTTCTGAACATTGACCA		
*Cytochrome b*	CB-J-10933	TCTTTTTGAGGAGCWACWGTWATTAC	386	Simon et al. (1994)
	CBN-11367	AATTGAACGTAAAATWGTRTAAGCAA		
*EF-1α*	Elongation factor forward	AGATGGGYAARGGTTCCTTCAA	418	Belshaw and Quicke (1997)
	Elongation factor reverse	AACATGTTGTCDCCGTGCCATCC		
*ND1*	ND1-F	ACTAATTCAGATTCTCCTTCT	447	Smith et al. (1999)
	ND1R	CAACCTTTTAGTGATGC		Smith and Kambhampati (1999)
*Wingless*	Wg550F	ATGCGTCAGGARTGYAARTGYCAYGGYATGTC	448–458	Wild and Maddison (2008)
	WgAbRZ	CACTTNACYTCRCARCACCARTG		

**Table 2.** **PCR amplification conditions for the seven genetic markers used in this study.** These optimised PCR conditions were applied to ensure consistent and high-quality amplification for downstream sequencing and phylogenetic analysis.

Gene	Initial denaturation	cycle			Final extension
		denaturation	annealing	extension	
*28S D2*	95℃ 3 min	32 cycles			72℃ 10 min
		95℃ 30 s	48℃ 30 s	72℃ 1 min	
*COI*	95℃ 5 min	35 cycles			72℃ 7 min
		94℃ 1 min	54℃ 1 min	72℃ 90 s	
*COII*	95℃ 3 min	33 cycles			72℃ 5 min
		94℃ 1 min	52℃ 1 min	72℃ 90 s	
*cytochrome b*	93℃ 5 min	40 cycles			72℃ 3 min
		92℃ 1 min	45℃ 1 min	72℃ 1 min	
*EF-1α*	95℃ 3 min	40 cycles			72℃ 5 min
		94℃ 30 s	48℃ 30 s	72℃ 90 s	
*ND1*	95℃ 3 min	35 cycles			72℃ 7 min
		95℃ 30 s	48℃ 1 min	72℃ 1 min	
*wingless*	95℃ 3 min	40 cycles			72℃ 7 min
		94℃ 1 min	46℃ 2 min	72℃ 2 min	
